# The Italian Portrait of Laboratory Information Systems in Pathology: The Ones We Have and the Ones We Would Like

**DOI:** 10.3390/jpm15110517

**Published:** 2025-10-31

**Authors:** Stefano Marletta, Marco Maria Baron, Vincenzo L’Imperio, Aldo Scarpa, Alessandro Caputo, Giuseppe Perrone, Francesco Merolla, Umberto Malapelle, Matteo Fassan, Angelo Paolo Dei Tos, Fabio Pagni, Albino Eccher

**Affiliations:** 1 Department of Biomedical Science, Humanitas University, 20072 Milan, Italy; stefano.marletta@humanitascatania.it; 2 Division of Pathology, Humanitas Istituto Clinico Catanese, 95045 Catania, Italy; marco.baron@humanitascatania.it; 3 School of Medicine and Surgery, University of Milano-Bicocca, 20126 Milan, Italy; vincenzo.limperio@unimib.it (V.L.); fabio.pagni@unimib.it (F.P.); 4 Department of Pathology, Fondazione IRCCS San Gerardo dei Tintori, 20900 Monza, Italy; 5 Section of Pathology, Department of Diagnostics and Public Health, University of Verona, 37134 Verona, Italy; aldo.scarpa@univr.it; 6 Pathology Department, University Hospital “San Giovanni Di Dio E Ruggi d’Aragona”, 84131 Salerno, Italy; alcaputo@unisa.it; 7 Operative Research Unit of Anatomical Pathology, Fondazione Policlinico Universitario Campus Bio-Medico, 00128 Rome, Italy; g.perrone@policlinicocampus.it; 8 Research Unit of Anatomical Pathology, Department of Medicine, Università Campus Bio-Medico, 00128 Rome, Italy; 9 Department of Medicine and Health Sciences “V. Tiberio”, University of Molise, 86090 Campobasso, Italy; francesco.merolla@unimol.it; 10 Department of Public Health, University Federico II of Naples, 80131 Naples, Italy; umberto.malapelle@unina.it; 11 Surgical Pathology and Cytopathology Unit, Department of Medicine-DIMED, University of Padua, 35128 Padua, Italy; matteo.fassan@unipd.it (M.F.); angelo.deitos@unipd.it (A.P.D.T.); 12 Pathology Unit, Department of Laboratory Medicine and Anatomical Pathology, AOU Policlinico di Modena, 41125 Modena, Italy; 13 Section of Pathology, Department of Medical and Surgical Sciences for Children and Adults, University Hospital of Modena, University of Modena and Reggio Emilia, 41125 Modena, Italy

**Keywords:** traceability, laboratory information systems, pathology workflow, data security

## Abstract

**Background**: In the evolving landscape of pathology, Laboratory Information Systems (LISs) have become essential tools for ensuring traceability, efficiency, and data security in diagnostic workflows. **Methods**: This study presents a comprehensive comparative analysis of three major LIS platforms used in Italian pathology laboratories in 2025: *Armonia* (Dedalus), *Pathox Web* (Tesi Group), and *WinSAP 3.0* (Engineering). Each system is evaluated across key parameters, including sample traceability, integration with hospital systems, digital reporting, user interface, and compliance with regulatory standards such as GDPR and ISO 15189. **Results**: *Armonia* stands out for its advanced integration capabilities, scalability, and support for digital pathology, making it ideal for large institutions. *Pathox Web* offers a balanced solution with strong usability and web-based accessibility, suitable for medium-sized laboratories. *WinSAP 3.0*, while more limited in modern features, remains a stable and cost-effective option for many facilities. This study emphasizes the strategic importance of selecting an LIS aligned with institutional needs, highlighting its role in enhancing diagnostic quality, operational safety, and future integration with artificial intelligence and automation. **Conclusions**: The findings support informed decision-making in LIS adoption, critically contributing to the management of scientific and economic data of pathology services in Italy.

## 1. Introduction

Pathological anatomy is a fundamental pillar of diagnostic medicine, providing crucial indications for the identification, staging, and therapeutic management of a wide range of diseases, particularly oncological ones. Within this field, the reliability and safety of the diagnostic process depend on the traceability of biological samples and the correct management of clinical and laboratory data, highlighted by international regulatory accreditation releases [1,2]. Laboratory Information Systems (LISs) have assumed a central role, becoming indispensable tools to ensure high standards of quality, efficiency, and safety [3], especially in the modern scenario of transition to digital pathology laboratory and networks [4,5,6,7]. Given the growing importance of software in the medical device sector, whether as standalone software or embedded in another medical device, an essential requirement for its use is validation according to the state of the art.

The laboratory workflow, divided into pre-analytical, analytical, and post-analytical phases, has benefited from the introduction of LISs capable of managing patient test requests and processing and storing information generated by laboratory equipment. Depending on the level of integration with hospital information systems, the LIS can manage the exchange of information with the Central Booking System (CUP), the Hospital Information System (HIS), the systems of individual hospital departments, the Electronic Patient Record (EPR), and the Personal Health Record (PHR). The potential for integration between these systems has been facilitated by the international initiative IHE (Integrating the Healthcare Enterprise) [8], which promotes the use of already defined standards in the medical field and coordinates them in a performant and advantageous manner to adapt them to the relevant healthcare scenario.

Standardization procedures are not only indispensable but also an opportunity to initiate reorganization processes and improve healthcare performance [9,10]. Their potential ensures increased productivity, reduced laboratory errors [11,12], and greater safety for healthcare professionals and the entire analytical process. The presence of a single health database containing unique, easily updatable records reduces the risk of patient misidentification and, in particular, offers the possibility of accessing previous test results, which is greatly useful in outlining the clinical picture of chronic patients [3].

This article seeks to conduct an in-depth comparative analysis of various LISs for pathology laboratories, with the goal of examining and comparing the potential of each system in terms of functionality, traceability, and management of the main phases of the diagnostic process. The article focuses on the most commonly used systems in Italy and how they support the different work phases, including macroscopic examination, inclusion, sampling, cutting, immunohistochemistry, test delivery, reporting, image scanning, archiving, and management of intraoperative procedures. The insights from this study will provide sector operators and business decision-makers with essential support in choosing the most suitable management system for pathology laboratories’ specific diagnostic and organizational needs, with a particular focus on service quality, data security, and ease of daily workflow management.

## 2. Context

Modern LISs have enabled the unique identification of samples through barcodes [13], the digitization of reports, the integration of clinical data via interoperable standards (such as HL7 and FHIR) [14,15], and the adoption of electronic signature tools. This has made it possible to precisely monitor every phase of the process, from sample collection to reporting, ensuring complete traceability, error reduction, increased timeliness, and compliance with data protection regulations (e.g., General Data Protection Regulation—GDPR) [16]. In today’s laboratory practice, management systems are configured as fully integrated platforms capable of supporting all operational and administrative activities while ensuring security, auditability, and transparency. Interaction with external devices—such as barcode printers, digital scanners, image acquisition systems, and digital storage devices—allows for automated management of the diagnostic cycle, significantly reducing the margin of error and improving overall efficiency.

**Figure 1:**
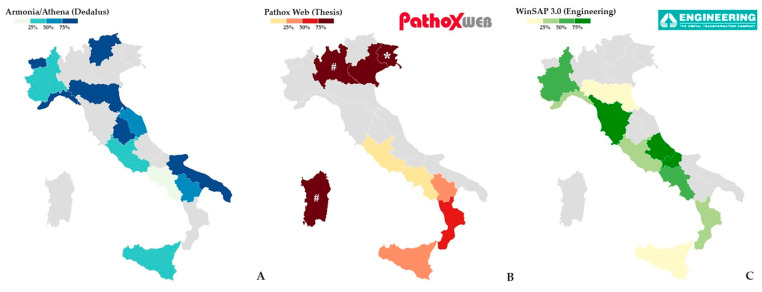
Graphical distribution of LIS in Italy regions: *Armonia*/*Athena *(*Dedalus*) (**A**), *Pathox Web *(*Tesi*) (**B**), and *WinSAP 3.0 *(*Engineering*) (**C**). LIS: laboratory information system. * Friuli-Venezia Giulia relies on Pathox Web just for the digital pathology module, while using a home-made solution for all the other tasks. # Lombardy and Sardinia laboratories will employ Pathox Web by the date of the publication of this article, following a regional market consultation. Data was collected through direct pathologists’ interviews and an online survey.

In the current Italian landscape, more than 90% of the major public and private pathology laboratories are equipped with one of the following three management systems: *Armonia*—or similar, like *Athena*—(Dedalus), *Pathox Web* (Tesi Group), and *WinSAP* 3.0 (Engineering) (Figure 1 and Table 1). These three systems differ in their IT architecture, modularity, integration capabilities with other hospital systems, support for Digital Pathology (DP), sample traceability methods, and quality control tools. To note, a minority of laboratories are haphazardly equipped with other company-provided or home-made LISs, generally provided with a less wide range of functionalities.

The following sections will compare the three major systems based on a series of key parameters that reflect the current needs of laboratories, including:

• Sample traceability at every stage of the pre-analytical, analytical, and post-analytical workflow;
• Integration with the HIS and external diagnostic devices;
• Support for digital reporting and electronic signatures;
• User interface and usability for pathologists and technicians;
• Image management and compatibility with digital pathology;
• Cybersecurity, audit trails, and regulatory compliance;
• System customization and scalability.

This comparative study aims to provide a useful tool to outline the minimum standards expected from a modern LIS for anatomical pathology, highlighting strengths and weaknesses with particular attention to user experiences in real-world contexts.

### 2.1. Backbone

#### 2.1.1. Armonia (Dedalus)

*Armonia* is a next-generation LIS developed by Dedalus Group (Milan, Italy), a leading European company in healthcare IT. This LIS is designed to natively support the entire laboratory workflow, including complex workflows such as those in pathology, with full integration into the Digital Health System. It is based on a modular client–server architecture, allowing high flexibility in configuration and scalability. It can be installed both on premises and on the cloud, utilizing modern programming languages like Java and SQL. The system enforces strict security policies: access via profiled credentials, support for strong authentication with smart cards, OTP, granular permission management, and operation audits. *Armonia*’s interface is designed to be configurable based on the user’s role: technician, pathologist, or administrator. The dashboard layout allows quick access to main tasks, notifications, and real-time activity monitoring.

#### 2.1.2. Pathox Web (Tesi)

*Pathox Web* is an information system for pathology developed by Tesi Group (Milan, Italy), an Italian company with extensive experience in healthcare IT. This web-native LIS is entirely designed to manage every phase of the diagnostic process in pathology in a structured and traceable manner, from clinical request to report archiving, including all technical-laboratory steps. *Pathox Web* is a browser-based application that relies on a centralized infrastructure in a Windows Server environment, compatible with SQL Server as the main database, using Angular for the web frontend, .NET 6 for the backend, and PostgreSQL for the database. Access is via a web browser, ensuring maximum usability on the hospital intranet. *Pathox Web*’s interface is modular and vertical: each system area (acceptance, technical processing, reporting, administration) is organized into tabs accessible via a left menu, with extensive use of colors to distinguish states (e.g., “in processing,” “waiting,” “completed”). Screens are designed for high readability, with quick search options and table customization. The system provides differentiated user profiles (e.g., technician, pathologist, manager), each with targeted access to necessary functionalities.

#### 2.1.3. *WinSAP 3.0* (Engineering)

*WinSAP 3.0* is a historical system for pathology developed in Italy and used in various public and private hospital facilities. It is an LIS initially designed for local use and operational simplicity. *WinSAP 3.0* is based on a client–server architecture with a relational database and traditional graphical user interfaces (Windows-based GUI). Access to the system is from fixed workstations connected to a local network (LAN). Although not web-based, it can interface with other information systems through integrative modules or HL7 bridges, albeit less dynamically than more modern LIS. The languages used are Visual Basic and Access/SQL databases. A recent update has also allowed web-based usage of the LIS. *WinSAP 3.0*’s user interface is functional but less modern. Screens are heavily oriented towards rapid keyboard input, reflecting the operational environment of the 1990s and 2000s. The learning curve is steep initially, but the system’s operational stability and predictability make it reliable in the long term.

## 3. Digital Workflow in Pathology and Comparison of Various LIS

**Figure 2:**
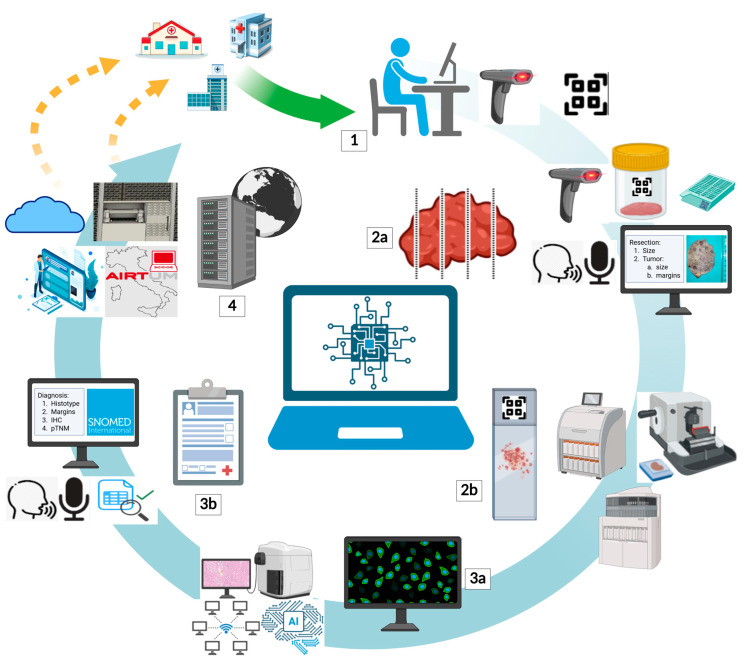
Role of the LIS throughout the pathology workflow. Sample acceptance (1): The LIS should be able to register samples collected from in- and outpatients’ services, especially for “hub-and-spoke” networks, by identifying them with unique codes. Grossing (2a): the LIS ought to guide pathologists during macroscopic assessment by codifying the unique sample code, printing bio-cassettes, integrating macroscopic images, supporting voice recognition, and allowing descriptions by pre-filled templates. Technical sample management (2b): to ensure reliable traceability, the LIS must be interfaced with blocks and slides’ unique codes during processing, embedding, sectioning, and staining. Whole slide imaging (WSI) visualization (3a): ideally, the LIS should crosstalk with slide scanners and WSIs viewer to let pathologists visualize and navigate digital slides, supporting integration with artificial intelligence-based tools and case sharing via online network services. Microscopic reporting and signing (3b): modern LIS are required to enable the creation of diagnostic templates and should allow voice recognition dictation, editing systems, and automatic link of diagnoses with systematized nomenclature codes (i.e., SNOMED). Storage (4): once the diagnosis is signed, it is down to the LIS to have the digital report sent to inpatients and outpatients’ physicians and to integrate it into electronic health repositories; furthermore, the LIS must also be interfaced with hardware and software responsible for the storage of blocks, physical, and glass slides.

The phases of the workflow in a modern pathology laboratory are fully trackable, contributing not only to improving diagnostic quality but also to meeting regulatory requirements for data security, clinical risk management, and medico-legal responsibility. The integration of barcodes, RFID, HL7 interfaces, and digital signature tools enables synergistic management between the laboratory, clinicians, and hospital systems. The technical and diagnostic workflow in pathology is structured into a sequence of integrated phases, each playing a crucial role in the quality of the ultimate result. Each phase is now supported and tracked by the LIS, ensuring control, consistency, and security throughout the diagnostic process. In this context, *Armonia*, *Pathox Web*, and *WinSAP 3.0* are three widely adopted solutions, each with specific features for managing these key steps. This complex workflow in pathology can be divided into four fundamental phases (Figure 2):

1. Request Registration and Sample Acceptance: entering the clinical request into the IT system, identifying the patient and associating the sample, verifying the correspondence between the request and the sent material, logistical registration, and starting internal traceability.
2. Grossing and Technical Processing: preparing the sample through fixation, sampling, paraffin embedding, sectioning, staining, and slide mounting.
3. Microscopic Reporting and Signing: microscopic observation by the pathologist, diagnosis formulation, report compilation, and validation with the application of a qualified electronic signature.
4. Archiving and Sending to the Requester: saving in the LIS, possible physical archiving of materials, and sending the report to the attending physician.

These phases represent the operational stage of a modern pathology laboratory. The efficiency, quality, and security of the entire process depend on the precision with which each phase is implemented, documented, and integrated into the information systems.

### 3.1. Sample Acceptance

#### 3.1.1. Armonia

*Armonia* allows acceptance through integration with scheduling and order entry systems, supporting barcode reading. However, the flexibility of configuration depends on external modules and the client’s infrastructure. It stands out for its strong customization capabilities in workflow management, ensuring high levels of traceability and automation. It features a modern, modular user interface designed to support both desktop devices and mobile and touchscreen solutions, enhancing usability in dynamic environments. It has a centralized acceptance worklist with the ability to filter samples based on clinical parameters (type of test, urgency, priority, etc.), optimizing request management. It integrates patient demographics with full compatibility with hospital ADT systems and health registries, ensuring real-time data consistency and updates. It uses an automatic printing system for customizable labels, which can be associated with different types of containers and operational phases, with barcode management for complete traceability. *Armonia* outdoes in high sample traceability from the moment they get to the laboratory, drastically reducing the risk of errors through intensive use of automatic coding and alerts for incongruent data. However, the initial configuration of workflows can be complex and may require specialized staff training.

#### 3.1.2. Pathox Web

It allows both automatic and manual acceptance with native integration with scheduling, order entry, and external systems. This program assigns a unique identifier, supports barcodes, and allows check-in directly from mobile devices thanks to its responsive interface. The user interface is entirely web-based, responsive, and user-friendly, adaptable to various devices and browsers. It can manage acceptance requests both directly in the laboratory and remotely (e.g., from clinical departments sending samples), optimizing time and resources. It synchronizes patient demographics in real-time with hospital information systems and electronic health records. It dynamically generates unique identification labels with the ability to associate multiple containers with a single request and supports on-demand printing from different workstations. Pathox Web’s greatest strength lies in its high operational flexibility, allowing for mass acceptance in high sample volumes. Its compatibility with HL7 and FHIR standards makes it easily integrable with other healthcare solutions. However, interface customization may have some limitations on less updated browsers or specific corporate configurations, sometimes requiring additional modules for implementing advanced functions.

#### 3.1.3. *WinSAP 3.0*

This software manages requests through manual entry or HL7 protocols; acceptance is divided into administrative acceptance and subsequent analytical acceptance, with separate operations ensuring precise control but requiring more manual effort. It utilizes standard label printing, with the option for integration with external industrial labeling systems. *WinSAP 3.0* ensures reliability even with high historical data volumes and is particularly appreciated in environments with experienced personnel due to its interface optimized for immediate use and check-in functionalities integrated with corporate and regional systems. However, the lack of automation in acceptance processes compared to more modern systems implies greater exposure to manual errors and a lower capacity for workflow customization.

### 3.2. Sample Traceability (Sampling, Embedding, Sectioning, Staining Slides)

#### 3.2.1. Armonia

It provides basic traceability of operational phases, but customization depends on the activated packages. The interface is modern, responsive, and suitable for touchscreen devices, facilitating quick interaction even on dedicated workstations. Macroscopic workflows are clearly structured, allowing immediate visualization of the sample’s status. *Armonia* implements an advanced traceability system: each sample is identified via barcode or QR code, uniquely associated with containers and embedded cassettes. Macroscopic descriptions are assisted by dynamically configurable templates, allowing guided entry based on the type of specimen. A standardized library of tissue and morphological terms is easily accessible. The platform natively integrates macroscopic image management, allowing direct photographic capture from digital devices with immediate saving in the clinical case. Images can be annotated and associated with individual cassettes. *Armonia* supports voice recognition systems like Dragon Medical or equivalents, enabling direct dictation of macroscopic descriptions. It is designed to integrate with automated sampling and photography systems (e.g., MacroPATH Milestone), allowing automatic data transmission and recording. A complete audit trail is implemented: every modification to the macroscopic description or sample data is recorded in detail for medical-legal and internal traceability purposes. For embedding, sectioning, and staining phases, the operator is guided step-by-step by an interactive digital checklist that prevents proceeding to the next phase without correctly completing the current one. Before the next phase, the barcode or RFID tag associated with the cassette must be scanned, and the system then requires confirmation of the association between the sample, the cassette, and the future paraffin block. If a scan is not performed or the code does not match, the system automatically stops the process, preventing identification errors. *Armonia* natively supports 2D barcodes and offers RFID support as an option. Every operation is tracked: who handled the sample, at what time, on which embedding station, and with which device identifier. The interface is optimized for touchscreen devices and is usable even with laboratory gloves. Operators have screens showing the status of cassettes to be embedded, sectioned, and stained, those already completed, and those awaiting processing. The system not only issues visual and auditory alerts in case of discrepancies but also requires the operator to resolve the anomaly before proceeding. Automatic operational blocks are provided if data mismatches are detected. *Armonia* allows a high degree of configurability of the embedding workflow, adapting to the internal operational protocols of each laboratory. It is also designed for direct integration with automated embedding stations, allowing real-time sample data transmission.

#### 3.2.2. Pathox Web

*Pathox Web* ensures comprehensive management of all processing phases (embedding, sectioning, staining, etc.), traceability with unique barcodes, and integration with MacroPathox and instrumental devices. It also guarantees robust traceability through the use of barcodes. The system tracks the main steps of the sample, from collection to delivery in the dissection room. Macroscopic descriptions are made using preconfigured forms, differentiated by sample type. Checklists and Systematized Nomenclature of Medicine Clinical Terms (SNOMED) standards [17] can be used, although not completely dynamically. The user may need to adapt to fewer flexible forms in some cases. Image management support is available but optional: the platform can integrate with image acquisition systems, but requires additional modules. *Pathox Web* can integrate voice recognition systems, although this integration is not native and requires custom configuration. Connection with macroscopic automation devices is possible but may require specific interfacing projects. *Pathox Web* maintains an audit trail of main operations. However, the level of detail in recording microscopic or descriptive changes is lower than *Armonia*’s. *Pathox Web* adopts a streamlined workflow based on configurable process events. The system guides the operator through a logical sequence of operations but allows greater decision-making freedom: the operator, for example, can force some operations in case of anomalies without completely blocking the system, provided the exception is documented. For embedding, sectioning, and staining blocks/slides, the system requires scanning the cassette barcode, but RFID integration is available only on request as an optional module. Sample tracking is automatic, although the audit trail is less detailed than *Armonia*’s. The interface is more technical and less touch-oriented than *Armonia*’s, but it is still easily accessible from tablets or PCs. In case of scanning or data association errors, the system issues a warning. However, the operator can choose to proceed forcibly by documenting the exception. This feature is useful in environments where maintaining some flexibility is necessary but could increase the risk of human error. *Pathox Web* is moderately customizable, with the ability to configure many aspects of the operational flow, still requiring advanced administration interventions. Integration with automated embedding instruments is available but often needs specific interfacing interventions.

#### 3.2.3. *WinSAP 3.0*

The system provides advanced management of laboratory workflows with real-time traceability. It integrates directly with laboratory instruments and process monitoring. Native support for the concept of a unified logical laboratory is included. The interface is more traditional, built on multiple windows, with a steeper learning curve for less experienced users and limited touch use. The system supports sample tracking with barcodes, but some container-cassette association operations must be managed manually. It does not natively support QR codes or RFID technologies. Macroscopic description management is based on free text fields and pre-set phrases. Dynamic templates and standardized classification support systems are not available. *WinSAP 3.0* does not integrate native image management: any macroscopic photos must be managed with external systems and manually uploaded. It does not natively support voice dictation: external software must be used in stand-alone mode. *WinSAP 3.0* does not provide direct integration with automatic sampling or photography systems: any connections must be custom-designed. The software records main events (e.g., sample data entry/modification), but the completeness level of tracking is lower than more recent standards, as required by UNI EN ISO 15189 certifications [18,19]. *WinSAP 3.0* presents a simplified and less structured management of embedding, sectioning, and staining phases compared to other software. The operator manually enters data related to the listed work phases, with the possibility of scanning the barcode associated with the cassette, but without a binding or highly controlled workflow. Traceability is present, although in an essential form, as the system records the operator and timestamp without performing strict cross-checks. It is possible to use 1D or 2D barcodes to identify cassettes; however, native support for RFID is not provided and can only be implemented through external components. The system alerts the operator in case of discrepancies but does not block operations. Anomalies are only reported via on-screen messages, leaving the operator fully responsible for managing the situation. *WinSAP 3.0* offers limited customization possibilities for the embedding workflow, as integration with automated machinery is possible but not native and requires manual adjustments or custom-developed additional modules.

### 3.3. Microscopic Reporting and Signing

#### 3.3.1. Armonia

The system allows for the automatic generation of reports using predefined and customizable templates. Reports can be pre-filled with data from other sources (e.g., RIS, PACS), reducing the risk of transcription errors. Reports can be tailored to the specific needs of the laboratory, enabling the use of specific templates for each type of examination (histology, cytology, special tests). Based on specific needs, each template can be configured with free or structured fields. *Armonia* includes quality control features that verify the completeness of the data entered in the report and its consistency with the clinical data present in the system. Automatic cross-checks are also provided to identify possible errors or inconsistencies. The system allows direct integration of generated reports into the electronic medical record system, ensuring centralized data management and easy access by attending physicians. *Armonia* offers a high level of customization of reports, useful for laboratories with complex diagnostic needs. The ability to easily integrate with other corporate modules (RIS, PACS) and hospital systems allows for centralized information management.

#### 3.3.2. Pathox Web

Being web-based, it allows access from any network-connected device, enabling professionals to draft reports even from remote locations. The system offers assisted reporting with a customizable editor, support for checklists, predefined phrases, SNOMED, ICD-O, pTNM, and ICD-10 coding. It provides multiple layout options, digital and remote signatures, with a wide range of predefined templates that can be used for report generation. The templates are structured to simplify data entry, reducing the risk of errors. *Pathox Web* lets the inclusion of images (such as microphotographs of samples), graphs, and other multimedia data within the reports, improving the quality and comprehensibility of diagnostic information. The system easily integrates data from RIS and PACS, allowing centralized management of all diagnostic and clinical information. Additionally, images or multimedia documents can be attached to the reports for a more complete diagnostic picture. Since the system is web-based, internet connectivity is essential for its proper functioning, and network issues can slow down or interrupt the reporting process. Advanced customization of templates and features is more limited compared to other software, especially in contexts requiring highly specific configurations.

#### 3.3.3. *WinSAP 3.0*

The software allows the generation of textual reports, with the possibility to insert comments, diagnoses, and links to multimedia files, such as images and tables. Recently, the software has been provided with browser accessibility, letting pathologists remotely sign diagnoses by logging in with personal ID digital usernames. The system includes automatic coding modules that associate each diagnosis with a standardized code, such as ICD-10 or SNOMED CT, simplifying the registration and coding of pathologies. It includes a quality control module that verifies the data entered in the report, avoiding possible errors and ensuring the accuracy of the reports. The system is known for its ease of use, even for less experienced users, thanks to a simple and intuitive interface. The system offers more limited customization options compared to other solutions, especially regarding the management of templates and workflows.

### 3.4. Data Storage, Data Security, Integration with Instruments and External Systems, Dashboards, KPIs, and Business Intelligence

#### 3.4.1. Armonia

It employs a centralized data structure based on an SQL database (such as SQL Server or Oracle). This approach allows for unified information management, with data stored in interconnected tables, ensuring data consistency and integrity. The system archives samples using unique identifiers (barcodes or QR codes), associated with all information related to the sample, the diagnostic tests performed, reports, and images. The advantage of a relational database is that it allows for advanced queries to obtain quick and precise responses during data retrieval. Each sample has a well-defined documentation path that follows every phase of the sample’s lifecycle, from initial registration to final diagnosis. In case of further collection of other biological samples, the systems easily allow the retrieval of the archived data whenever.

Security is a crucial aspect of *Armonia*, which implements various measures to protect sensitive data. Data is encrypted both in transit (during transmission between devices) and at rest, to protect information from unauthorized access. It uses a multi-level authentication system to control who has access to which data. Users can be divided into groups with well-defined roles, each having access only to the information necessary for their functions. Every operation performed on the system is logged, creating a trace of all data modifications. This is essential for ensuring transparency and traceability of activities. An automatic data backup system is implemented, occurring regularly, with data stored in secure environments, with the possibility of backing up to multiple servers or in the cloud, to ensure no data loss in case of hardware failures. The system is designed to minimize downtime and ensure service continuity. Redundancy is guaranteed by a database and hardware-level architecture, allowing the system to function even if one of the servers fails, without compromising data integrity. *Armonia* complies with major international standards, such as UNI EN ISO 15189 (which sets requirements for medical laboratories) and GDPR. The system implements audit trail functions to track every data modification, a fundamental aspect for transparency and compliance with privacy and data protection regulations.

#### 3.4.2. Pathox Web

This program also relies on a relational database to store all information related to samples, tests, and reports. However, the main difference lies in its integration with other systems used by laboratories, as it can be integrated with image archiving systems (PACS) or electronic medical records, facilitating data exchange and interoperability between different software platforms. This integration allows for centralized data management, reducing the risk of data transcription errors and improving service quality. The system uses advanced encryption technologies, with multi-factor authentication (MFA) to ensure that only authorized users can access sensitive data. In addition to encryption and authentication, it also features a role-based access control system, assigning different privileges based on user functions and responsibilities. Stored data is protected against unauthorized access and accidental loss through stringent security measures. *Pathox Web* performs daily data backups and offers the possibility of storing data on external servers or in the cloud. This approach not only reduces the risk of data loss but also ensures that, in case of hardware failures, the system can be quickly restored without compromising operational continuity. The retrieval of data is quickly permitted whenever other samples belonging to the patient are collected. The visualization of reports and images is centralized and easily accessible, with a system that directly integrates diagnostic images within textual reports. *Pathox Web* complies with UNI EN ISO 15189 standards and GDPR provisions. An audit trail system is provided to track every data modification and ensure that the laboratory is always compliant with data protection regulations.

#### 3.4.3. *WinSAP 3.0*

Like the other systems, *WinSAP* *3.0* uses an SQL relational database to store sample and report data. The system is highly configurable and can be adapted to the specific needs of the laboratory. This includes customizing sample management, adding specific fields for diagnostic needs, and integrating with other software applications used in the laboratory. It ensures data protection through encryption, both during transfer and at rest. It also implements a role-based access control system that defines who can access or modify certain data. The system performs automatic daily backups and allows data to be stored in the cloud or on external servers and eventually retrieved whether further biological samples are collected. Additionally, *WinSAP* *3.0* provides data replication across multiple servers to ensure operational continuity in case of failures. *WinSAP 3.0* complies with UNI EN ISO 15189 and GDPR regulations and provides tools for monitoring data changes through an audit trail system, which allows tracking all performed operations.

## 4. Discussion and Future Directions

Traceability in pathology today represents not only a technical-operational requirement but also an ethical and clinical safeguard. In a context where patient safety, diagnostic quality, and medico-legal responsibility are central to the care pathway, the LIS emerges as an indispensable tool. Through it, every biological sample can be tracked at every stage from reception to reporting with precision, reliability, and documentary transparency. International agencies recognize the implementation of a robust LIS as essential for demonstrating the operational efficiency and safety of pathology laboratories. In this context, one of the critical requirements for achieving College of American Pathologists (CAP) accreditation is the presence of an up-to-date LIS [1]. Such a system must support key functionalities, including system security protocols; error detection mechanisms; auto-verification processes; and reliable transmission, retrieval, and preservation of data, among others.

**Figure 3:**
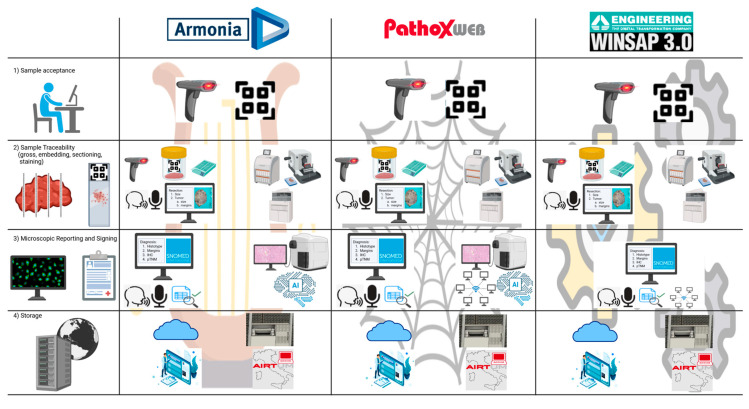
Comparison of functionalities provided by the three main LIS commonly used in Italy regarding the key phases of pathology laboratory workflow: sample acceptance, sample traceability, microscopic reporting and signing, and storage. Refer to Figure 2 for the icons’ meaning.

In today’s pathological anatomy laboratory, the sample acceptance phase constitutes the first critical junction of the diagnostic flow [9]. It must ensure the unique identification of the received material, its accurate registration, and the initiation of a traceable process in all its phases, as required by reference standards such as UNI EN ISO 15189 [18,19]. In this view, the use of a suitably customized and constantly updated LIS represents a strategic choice: investing in a good LIS means investing in the quality of care. The choice of the most suitable LIS management system must be based on a careful evaluation of the clinical and organizational needs of the laboratory, the available technologies, and future evolution prospects. In this comparative analysis (Figure 3 and Table 2):

• *Armonia* stands out for its completeness and modernity. Its main advantages include high scalability, strong integration with other clinical systems, a flexible interface, and continuous support from the manufacturer. However, the licensing and maintenance costs are significant, and the initial implementation can be complex.
• *Pathox Web* represents a good compromise between functionality and simplicity. Its strengths include a user-friendly interface, browser accessibility, and a good quality/price ratio. On the other hand, it is less performant in high-volume contexts or where integration with advanced diagnostic tools is required.
• *WinSAP 3.0* remains an economical and stable choice for small-sized contexts where the adoption of new technologies is limited. Despite a bit outdated interface, it has been recently updated with advanced functionalities such as online remote accessibility.

The analysis particularly considered the acceptance phase of histological samples, evaluating fundamental aspects such as the ease of use of the interface, demographic integration with other clinical systems, labeling quality, and the ability to prevent operational errors. However, the differences between the three systems also emerge in subsequent steps of the pathology workflow, such as macroscopic sampling, inclusion, cutting, staining, reporting, and digital signing.

In summary, this work emphasizes how the correct adoption of an LIS in pathological anatomy is not just a technological choice but a strategic decision to protect diagnostic quality [20], clinical safety, and organizational efficiency. Only a system capable of ensuring full traceability, adaptability, and compliance can truly meet today’s challenges and anticipate those of tomorrow. These considerations are crucial in the modern landscape of a digital transition in pathology, where fully tracked integration with software [21], scanners [22], monitors [23], and reporting [17,24,25] is the key to ensuring safe data management, especially in hub-and-spoke organized networks [4,5]. For large hospital and university structures, where interdepartmental interconnection, process digitization, and large data volume processing are the order of the day, *Armonia* represents a suitable choice. Its ability to adapt to complex workflows and integrate with other systems makes it ideal for these contexts. For medium-sized laboratories or private structures that desire a functional solution, remotely accessible, and with a good integration base, *Pathox Web* can offer the best balance between costs, functionality, and ease of use. For small laboratories or clinics with limited needs, which do not require strong digitization or advanced integration with other systems, *WinSAP* can be an economical and sufficient choice, provided a lower adherence to modern standards is accepted.

The future of LIS for pathology pushes towards greater integration with artificial intelligence and automation systems, and the secure sharing of data on a national and international scale. In this view, automation solutions keep emerging on the market for the pre-analytical [26], analytical [9], and post-analytical phases [17,27], easing the workflow, reducing costs, human errors, and turnaround times. Fundamental will be the adherence to common standards (e.g., HL7 and FHIR) [15] for total interoperability, the use of blockchain for process certification, complete digitization [6], including macroscopic with augmented visual support, predictive automation of diagnoses, and reporting processes. As for this latter, LIS’s integration with the digital signing of pathology reports should be accompanied by the automatic calculation of the diagnostic “weight” of each case. In the Italian healthcare system, laboratories are reimbursed based on the number and complexity of diagnoses provided for both inpatient and outpatient services. Each diagnosis is assigned a specific score—or “weight”—reflecting its complexity and the resources required, as defined in the SIAPEC Pathology Services Nomenclature. This point-based system, updated in 2025 [28], offers a comprehensive catalog of diagnostic procedures available to patients and their referring physicians. Such classification is of paramount importance not only for public hospitals, but also for private institutions, where an efficient reimbursement system is vital to couple the best patient management possible with the organization’s needs. Thus, by enabling automatic calculation of SIAPEC scores, LISs could also serve as valuable tools for budget planning and market benchmarking in both public and private healthcare settings, based on the range of services provided in histopathology, cytopathology, immunohistochemistry, and molecular pathology. In this context, the role of LISs in tracking molecular pathology has been widely underestimated. However, the disruptive advancements of diagnostics in this field, along with the increasing availability of highly automated kits [29] and robotic systems [9], are progressively leading to a huge increase in the volume of molecular services and associated reimbursement requests directed to pathology laboratories. As a result, the integration of current LIS with both manual and automated molecular pathology workflows is essential for the accurate management and traceability of this sensitive data.

To note, it is important to acknowledge certain limitations of this work. Firstly, although the analysis was informed by a critical review of available datasheets and real-world practices, the evaluation of each LIS functionality reflects the authors’ personal perspectives. As such, other users may have different experiences. Secondly, the data on the distribution of LIS across Italian regions (Figure 1 and Table 1) were collected through direct interviews and an online survey. Consequently, some information—particularly from smaller private pathology laboratories—may be incomplete or missing. Lastly, while we chose to focus on the three most widely used LIS versions, this was not intended to undervalue the capabilities of other, less commonly used or custom-built systems.

Looking ahead, the evolution of LIS platforms must address emerging challenges in interoperability, scalability, and AI integration. As highlighted by published literature, the digital transformation of pathology requires systems that not only support traceability and compliance but also enable seamless integration with AI-driven diagnostic tools and national data-sharing infrastructures [6]. Moreover, the incorporation of blockchain technologies for process certification and predictive analytics for diagnostic weighting could redefine the role of LIS from passive data repositories to active decision-support systems [6].

Future research should explore standardized benchmarking frameworks for LIS performance across diverse laboratory settings, which remain underrepresented despite their growing relevance. Such efforts will be essential to guide procurement decisions, foster innovation, and ensure equitable access to high-quality digital pathology infrastructure.

## 5. Conclusions

In conclusion, the management systems already analyzed today position themselves as pioneering tools in this direction and constitute a solid foundation on which to build the pathology laboratory of the future. In the present work, we have explored the functionalities of the three most commonly employed LISs in Italy. Our analysis outlines how the described systems are all valuable: *Armonia* for its full integration capabilities, *Pathox Web* for its web-based accessibility, and *WinSAP 3.0* for its cost-effectiveness. Indeed, the choice of an LIS should be tailored to the specific needs of each laboratory. These considerations notwithstanding, an LIS ensuring physicians a fully traceable workflow indeed represents a crucial tool to collect key scientific and economic information. While our study focuses on the Italian landscape, the main emerging issues do carry international relevance. Apart from facilities already equipped with a full-digital workflow, the majority of pathology services in Europe, America, and Asia still have to deal with similar concerns, especially regarding traceability, interface with tools, and storage. These data are the basis for building a proper laboratory organization and providing patients with the safest and most tailored management possible.

## Figures and Tables

**Table 1:** Distribution of LIS in the main public and private pathology laboratories in Italy.

	LIS, *n* (%)				
**Region**	Armonia	Pathox	WinSAP	Others/home-made	Overall
Aosta Valley	1 (100%)				1
Piedmont	2 (33%)		4 (67%)		6
Lombardy ^#^		32 (100%)			32
Veneto		12 (92%)		1 (8%)	13
Trentino-South Tyrol	2 (100%)				2
Friuli-Venezia Giulia *		6 (100%)			6
Liguria	3 (75%)		1 (25%)		4
Emilia-Romagna	8 (80%)		2 (20%)		10
Tuscany			7 (100%)		6
Umbria	4 (100%)				4
Marche	2 (67%)			1 (33%)	3
Lazio	11 (42%)	3 (12%)	8 (31%)	4 (15%)	26
Abruzzo			7 (100%)		7
Campania	1 (6%)	2 (11%)	11 (61%)	4 (22%)	18
Molise			2 (100%)		2
Basilicata	2 (67%)	1 (33%)			3
Apulia	11 (100%)				11
Calabria		2 (50%)	1 (25%)	1 (25%)	4
Sicily	9 (43%)	9 (43%)	1 (4%)	2 (10%)	21
Sardinia ^#^		9 (100%)			9
**Overall**	**55 (29%)**	**76 (41%)**	**44 (23%)**	**13 (7%)**	**188 (100%)**

Abbreviations: LIS: laboratory information system. * Friuli-Venezia Giulia relies on *Pathox* just for the digital pathology module, while using a home-made solution for all the other tasks. # Lombardy and Sardinia laboratories will employ *Pathox* by the date of the publication of this article, following a regional market consultation. Data was collected through direct pathologists’ interviews and an online survey.

**Table 2:** Comparison of functionalities provided by the three main LIS commonly used in Italy.

	*Armonia* (Dedalus)		*Pathox Web* (Thesis)		*Winsap 3.0* (Engineering)	
1. Sample Acceptance						
Unique code identification	Yes		Yes		Yes	
						
2. Sample Traceability						
Codifying unique sample code	Yes		Yes		Yes	
Printing biocassettes and slides	Yes		Yes		Yes	
Integrating macroscopic images	Yes		Yes		No	
Voice recognition dictation	Yes		Yes		No	
Prefilled gross description templates	Yes		Yes		Yes	
Interface with tools for embedding, cutting, and staining	Yes		Yes		Yes	
						
3. Microscopic reporting and signing						
Crosstalk with scanners, WSI viewers, and AI support	Yes		Yes		No	
Online network	No		Yes		Yes	
Creation of diagnostic templates	Yes		Yes		Yes	
Voice recognition dictation	Yes		Yes		Yes	
Editing services	Yes		Yes		Yes	
Automatic SNOMED link	Yes		Yes		Yes	
						
4. Storage						
Report integration into electronic health repositories	Yes		Yes		Yes	
Interface with hardware and software archives	Yes		Yes		Yes	

Abbreviations: LIS: laboratory information system, WSI: whole slide imaging, AI: artificial intelligence, SNOMED: Systematized Nomenclature of Medicine Clinical Terms; Green: the availability of specific functions for each system; Red: non-availability of specific functions for each system.

## Data Availability

No new data were created or analyzed in this study. Data sharing is not applicable to this article.
